# Investigation of miRNA and mRNA Co-expression Network in Ependymoma

**DOI:** 10.3389/fbioe.2020.00177

**Published:** 2020-03-19

**Authors:** Feili Liu, Hang Dong, Zi Mei, Tao Huang

**Affiliations:** ^1^Department of Neurosurgery, Huashan Hospital, Fudan University, Shanghai, China; ^2^Shanghai Institute of Nutrition and Health, Shanghai Institutes for Biological Sciences, Chinese Academy of Sciences, Shanghai, China

**Keywords:** co-expression network, ependymoma, miRNA, WGCNA, miR-15a, miR-24-1

## Abstract

Ependymoma (EPN) is a rare primary tumor of the central nervous system (CNS) that affects both children and adults. Despite the definition and classification of distinct molecular subgroups, there remains a group of EPNs with a balanced genome, which makes it difficult to predict a prognosis of patients with EPN. The role of miRNA-mRNA network on EPN is still poorly understood. We assessed the involvement of miRNA-mRNA pairs in EPN by applying a weighted co-expression network analysis (WGCNA) approach. Using whole genome expression profile analysis followed by functional enrichment, we detected hub genes involved in active proliferation and DNA replication of nerve cells. Key genes including CYP11B1, KRT33B, RUNX1T1, SIK1, MAP3K4, MLANA, and SFRP5 identified in co-expression networks were regulated by miR-15a and miR-24-1. These seven miRNA-mRNA pairs were considered to influence not only pathways in cancer and tumor suppression process, but also MAPK, NF-kappaB, and WNT signaling pathways which were associated with tumorigenesis and development. This study provides a novel insight into potential diagnostic biomarkers of EPN and may have value in choosing therapeutic targets with clinical utility.

## Introduction

Ependymomas (EPN) are rare neuroepithelial primary tumors of the brain and spinal cord that occurs in both children and adults. EPNs in childhood arise most often within the posterior fossa (PF) than supratentorial brain (ST), while spinal cord EPNs occur more frequently in adulthood. These neoplasms are the third most common central nervous system (CNS) tumors in children, accounting for 6–12% of brain tumors in childhood, while making up 1.8% of all primary tumors of the CNS (Ostrom et al., 2015, 2016). Traditionally, EPNs have been histologically classified as subependymomas and myxopapillary ependymomas (WHO grade 1), classic ependymomas (WHO grade 2), RELA-fusion positive (WHO grade 2 or 3), and anaplastic ependymomas as a high grade type (WHO grade 3) (Louis et al., 2016). It is particularly important to dig out a universal biomarker that can represent characteristics of EPN, which reflected distinct features. Currently, treatment for EPNs remains as maximal surgical resection combined with focal radiotherapy, which severely affects the growth and development of patients, especially children. The 10-year overall survival (OS) is 64% in children and ranges from 70 to 89% in adult patients with EPNs (Ostrom et al., 2015). The 5-year survival rate decreases to 42–55% in infants with EPNs (Gatta et al., 2014). It is becoming clear that to predict patient survival based on histopathologic features is challenging for ependymal tumors. Finding new molecular biomarkers and revealing molecular mechanisms will be more effective in guiding us to choose appropriate treatment strategies.

The microRNAs (miRNAs), small non-coding RNAs of approximately 22 nucleotides, fine-tune gene expression by binding to the 3′-UTR of the target mRNAs post-transcriptionally, causing gene silencing (Bartel, 2004). Previous studies reported a large group of mammalian mRNAs regulated by miRNAs (Friedman et al., 2009). It was shown that miRNAs could affect nearly one third of human genes (Bartel, 2009; Friedman et al., 2009) while a single mRNA could be targeted by various miRNAs, indicating that the gene regulation function of miRNA was involved in complex networks (Pan et al., 2018). Aberrant expression of miRNAs has been identified in many types of cancer (Pan et al., 2019a; Pan and Shen, 2019). Co-expression network analysis of miRNA and mRNA has attracted more attention in recent years, as it is believed to be one of the predominant regulatory types of gene expression (Liu and Yang, 2018). Thus, identification of miRNA–mRNA pairs and discovering the regulation network may potentially uncover useful cancer biomarkers. Recent research has revealed miR-15a and miR-24-1 are up-regulated in EPNs relapsed and deceased cases when compared to both the clinical remission cases and survivors, and may serve as a candidate of inferior prognostic molecules in children with EPNs (Braoudaki et al., 2016). As important regulators of life activities, miRNAs are closely related to cancer (Hayes et al., 2014) through its altered expression levels that affect the content of downstream genes (Esquela-Kerscher and Slack, 2006). It is necessary to detect both miRNA and associated mRNA during the diagnosis process, thereby increasing the reliability of the diagnosis. Identified co-expression miRNA-mRNA pairs in the current study can be applied as targets for clinical co-detection of ependymoma.

Weighted co-expression network analysis (WGCNA) can find co-expressed genes by calculating gene connectivity, and identify candidate biomarkers from a large number of gene sets rather than a small number of differentially expressed gene sets (Hu et al., 2012; Chang et al., 2013; Yang et al., 2018). It is a systematic biological method commonly used in current oncology research, and is valuable for mining prognostic markers involved in brain tumors. *In silico* technology greatly improves the efficiency of our research on molecular mechanisms and biological functions (Li et al., 2019b; Pan et al., 2019b; Yuan et al., 2019). Theoretical models of system biology provide reliable support for finding mechanisms of molecular interaction (Chen et al., 2017, 2018, 2019; Liu et al., 2017b; Li et al., 2018). The large data sets of both mRNA and miRNA expression profiles in the same patient could help discover the molecular mechanisms of miRNA-mRNA dysfunctions (Huang et al., 2015, 2016; Chen et al., 2016; Liu et al., 2017a; Zhou et al., 2019). In this study, WGCNA is used to identify the potential significant miRNAs and mRNA associated with diagnosis and prognosis of EPN, which is key for advancing our understanding on tumor progression of EPN and choice of optimal treatment strategies.

## Materials and Methods

### Dataset

The paired mRNA expression and miRNA profiles of 64 ependymoma patients were downloaded from NCBI Gene Expression Omnibus (GEO) with accession number GSE21687 (Wani et al., 2012). The 64 samples were all ependymoma primary tumors. There were 25 female and 39 male samples. The ages of youngest and oldest patients were 0.4 and 59 years, respectively. The median and mean ages were 8 and 13.2 years, respectively. Most of the samples were young patients. Within the miRNA profiles, there were 799 miRNAs that were measured with Agilent-019118 Human miRNA Microarray 2.0 G4470B (miRNA ID version). Within the 799 microRNAs, there were 723 human microRNAs. The mRNA profiles of 54,675 probes were measured with Affymetrix Human Genome U133 Plus 2.0 Array. For the probes corresponding to the same gene, we averaged their values to gene expression levels. To make the expression levels comparable between samples, we did quantile normalization (Ritchie et al., 2015). Finally, a total of 20,502 human mRNAs and 723 human miRNAs were used for downstream analysis.

**FIGURE 1:**
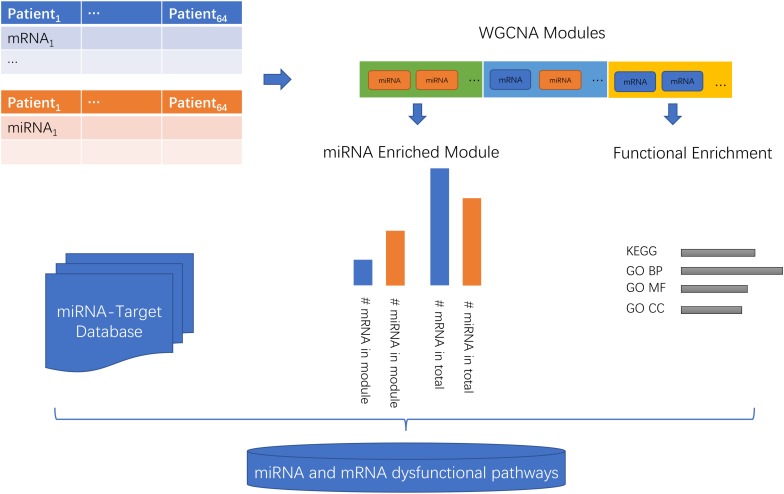
The workflow of the miRNA and mRNA co-expression network analysis. First, the miRNA and mRNA profiles of the same patients were combined. Then, the WGCNA network was constructed and the genes/miRNAs were clustered into modules. Then, the KEGG (Kyoto Encyclopedia of Genes and Genomes) and GO (Gene Ontology) Biological Progress (BP), Molecular Function (MF), and Cell Component (CC) functions of each module were investigated. Then, the enrichment of miRNAs in each module was evaluated using a hypergeometric test which compared the proportions of miRNAs in this module and in the background of total mRNAs and miRNAs. Lastly, with the help of the miRNA-target database, we inferred the miRNA and mRNA dysfunctional pathways.

The workflow of the miRNA and mRNA co-expression network analysis was shown in Figure 1. First, the miRNA and mRNA profiles of the same patient were combined. Then, the WGCNA network was constructed and the genes/miRNAs were clustered into modules. Then, the KEGG (Kyoto Encyclopedia of Genes and Genomes) (Kanehisa and Goto, 2000) and GO (Gene Ontology) Biological Progress (BP), Molecular Function (MF), and Cell Component (CC) (Gene Ontology, 2015) functions of each module were investigated. Then, the enrichment of miRNAs in each module was evaluated using a hypergeometric test which compared the proportions of miRNAs in this module and in the background of total mRNA and miRNAs. Lastly, with the help of the miRNA-target database, we inferred the miRNA and mRNA dysfunctional pathways.

### Co-expression Network Analysis

Screening hub genes and detecting co-expression of miRNA-mRNA pairs were performed by WGCNA, which is an R package for analyzing a weighted correlation network (Langfelder and Horvath, 2008). Appropriate soft-threshold power was selected to ensure co-expression network fitting scale-free topology. Associated genes were clustered based on dissimilarity of unsigned topological overlap matrix (TOM). Finally, we identified network modules and the genes/miRNAs within them.

### Functional Enrichment Analysis of Module Genes

We performed functional enrichment analysis on clustered genes in each module to detect the biological function of co-expressed hub genes. Gene Ontology (GO) terms included three parts: Biological Progress (BP), Molecular Function (MF), and Cell Component (CC). These were downloaded from the Gene Ontology database^1^ and the KEGG (Kyoto Encyclopedia of Genes and Genomes) pathways were downloaded from the KEGG database^2^. Hypergeometric distribution test (Li et al., 2019a; Pan et al., 2019b; Zhang et al., 2019) was applied to detect enrichment terms and *p*-values were corrected by False Discovery Rate (FDR) methods with a cutoff FDR < 0.05. Significantly enriched entries in all modules were sorted in ascending order by FDR value.

### Identification of miRNA-Enriched Modules

We used a hypergeometric test to identify the miRNA-enriched modules. For each module, we counted the number of members (genes and miRNAs) and the number of miRNAs and compared them with the number of all genes and miRNAs and the number of all miRNAs. Based on these numbers, we can calculate the statistical significance of miRNA enrichment in this module using a hypergeometric test. The modules with FDR < 0.05 were considered as miRNA-enriched modules.

### Detection of Target Genes of miRNAs

Interested miRNAs were searched against miRanda^3^, PicTar^4^, TarBase^5^, MirTarget^6^, miRBase^7^, and TargetScan^8^ databases to detect target genes of miRNAs. All records in the databases were retained and merged into a set of predicted miRNA target genes.

## Results

### Construction of Co-expression Networks for miRNAs and mRNAs

**FIGURE 2:**
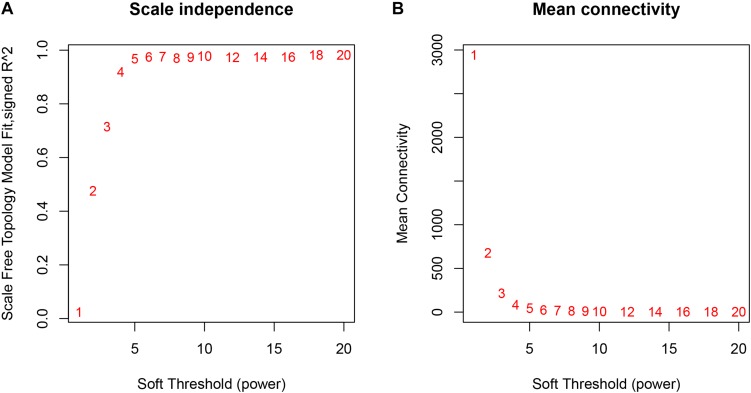
The relationship between Soft Threshold (power) and network properties. **(A)** The relationship between Soft-Threshold (power) and Scale Free Topology; **(B)** the relationship between Soft-Threshold (power) and Mean Connectivity. When Soft-Threshold (power) was five, the Scale Free Topology (R^2^) was 0.967 and Mean Connectivity became stable. Therefore, we setted Soft-Threshold (power) to be five.

**FIGURE 3:**
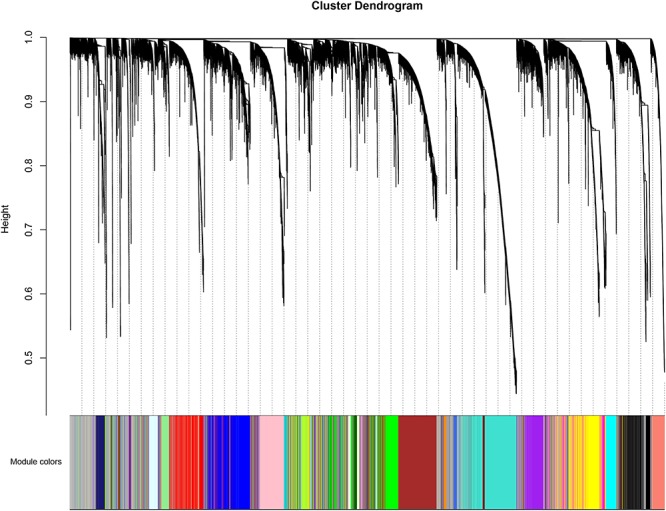
The cluster Dendrogram of the WGCNA co-expression network. The genes and miRNAs were clustered in 38 modules. Each module was marked with one color. Except for the gray module, which included many un-classified members, the turquoise module contained a maximum 1,943 members, while a minimum of 33 members were included in the skyblue3 module. In addition, we found 31 modules contained at least one miRNA and the maximum amount of 297 miRNAs were in the midnight-blue module.

The weighted gene co-expression network was constructed from 20,502 mRNAs genes and 723 miRNAs using the WGCNA approach. In our research, soft-thresholding power was set to be five to ensure the scale-free topology of the network (Figure 2). In Figure 2, the R^2^ meant how well the liner regression model fit for the association between degree and the log of the number of nodes with the corresponding degree. In scale-free network, the degree and the number of nodes with a corresponding degree has a power law relationship. Therefore, the degree and the log of the number of nodes with a corresponding degree has a linear relationship and the R^2^ of the linear regression model can be used to check how well the network fits the scale freeness. When soft-thresholding power was set to be five, the R^2^ was 0.967. A total of 38 modules were detected in this network and their relationship was shown in a cluster dendrogram (Figure 3). The number of members in different modules varies widely. The members of each module were listed in Supplementary Table S1. Beside the gray module which included many un-classified members, turquoise module contained the maximum 1,943 members, while the minimum 33 members were included in skyblue3 module. In addition, we found 31 modules contained at least one miRNA and the maximum amount of 297 miRNAs were in the midnight-blue module.

### Functional Enrichment Analysis of Modules

To investigate the functions of classified modules, we used a hypergeometric distribution test to analyze KEGG and GO enrichment on each clustered module (Supplementary Table S2). After FDR correction, enriched KEGG pathways with adjusted *p*-values (FDR), in which less than 0.05 were retained and sorted into ascending order. It was found that genes in the red module significantly enriched in cell cycle and DNA replication pathway (FDR = 6.61 × 10^–26^ and 5.23 × 10^–13^, respectively), which were important processes during cell proliferation. The synaptic vesicle cycle pathway and glutamatergic synapse pathway was remarkably enriched in turquoise module (FDR = 1.03 × 10^–14^ and 2.97 × 10^–11^, respectively), reflecting genes in such modules played important roles in the nervous system.

The Gene Ontology (GO) enrichment analysis was performed on all modules from three aspects: biological process, molecular function, and cellular component (Supplementary Table S2). Top terms in three groups were mitotic cell cycle process (GO: 1903047, FDR = 1.31 × 10^–61^), nucleic acid binding (GO: 0003676, FDR = 2.37 × 10^–30^), and synapse part (GO: 0044456, FDR = 1.71 × 10^–81^), which indicated that these co-expressed gene hubs were closely related to proliferation and genome replication of nerve cells.

Beside the KEGG and GO functional annotations, we enriched the modules with reported multi-omics ependymoma signatures: the 51 gene expression signatures of ependymoma survival from Supplementary Table 6 of Wani et al. (2012), and the validated 632 amplification and deletion genes of ependymoma from Supplementary Tables 5a,b of Johnson et al. (2010). For the 51 survival gene expression signatures, they were significantly enriched onto the brown and cyan modules with hypergeometric test FDR of 8.54E-07 and 0.000285, respectively. The brown module included 18 survival gene expression signatures (AGBL2, CASC1, CCDC81, DNAH9, DNAI1, EYA4, F5, GLB1L, IQCA1, IQCH, LRRC23, MYH15, MYLK3, NTS, SHANK2, SPAG6, TSNAXIP1, and WDR78) and the cyan module included 7 survival gene expression signatures (ANGPTL4, CHI3L2, ITGA5, SERPINE1, TAGLN, TGFBI, and VEGFA). For the 632 genes with validated amplification in ependymoma, they were significantly mapped onto the blue module with hypergeometric test FDR of 0.0319 and there were 61 overlapped genes. For the 728 genes with validated deletion in ependymoma, they were marginally significantly mapped onto the light cyan module with a hypergeometric test FDR of 0.0770 and there were 18 overlapped genes. It can be seen that the modules were associated with ependymoma survival and DNA copy number alterations (CNAs).

### Comparison With Reported Brain Gene Co-expression Modules

We compared our modules with the modules reported by Radulescu et al. (2018), in which the network was constructed based on the RNA-Seq data of 90 controls and 74 schizophrenia samples using WGCNA. The hypergeometric test FDR was calculated to evaluate the overlap significance between our modules and the modules reported by Radulescu et al. The results were given in Supplementary Table S3. All the modules reported by Radulescu et al. can be mapped onto our modules (hypergeometric test FDR smaller than 0.05); meanwhile, within the 38 modules identified by us, 21 modules can be mapped onto the modules reported by Radulescu et al. (hypergeometric test FDR smaller than 0.05). These results suggested that the main structure of the brain gene co-expression network was stable across different conductions and datasets, but we still got some ependymoma specific modules.

### Comparison With the Modules of Mouse Ependymoma Model

To investigate whether the ependymoma modules are conserved, we downloaded the mRNA expression profiles of 196 mouse ependymoma models from NCBI Gene Expression Omnibus (GEO) by accession number GSE21687. Similarly, we constructed their co-expression network using WGCNA and identified 16 modules (Supplementary Table S4). The hypergeometric test FDR was calculated to evaluate the overlap significance between our human modules and the mouse modules. The results were given in Supplementary Table S5. 13 out of 17 mouse modules can be mapped onto our human modules with hypergeometric test FDR smaller than 0.05, and 25 out of 38 human modules can be mapped onto the mouse modules with hypergeometric test FDR smaller than 0.05. These results suggested that the co-expression network was conserved across species.

### Identification of miRNA Enriched Modules

As mentioned above, 31 out of 38 classified modules possessed miRNAs. We further calculated percentages of miRNAs in total genes in corresponding modules to detect miRNAs-enriched functional modules. Five modules with hypergeometric test FDR smaller than 0.05 were considered to be miRNA-enriched modules (Table 1). More than half of the genes included in midnight-blue and royal-blue modules were miRNAs (84 and 53%, respectively). Furthermore, proportions of miRNAs in dark-turquoise (39%), yellow-green (35%), and saddle-brown (15%) modules surpassed the remaining modules (<10%), suggesting that several miRNAs-enriched gene hubs were interaction networks mediated by miRNAs. Meanwhile, the miRNA under enriched modules with hypergeometric test FDR smaller than 0.05 were listed in Table 2. These modules include less miRNAs than expected when considering the sizes of these modules. In other words, these modules were dominated by mRNAs. There were 17 such miRNA under enriched modules.

**TABLE 1:** The miRNA enriched modules.

**Module name**	**FDR**	***P-*value**	**Module size**	**Expected number of miRNAs**	**Number of miRNAs**
Midnightblue	0	0	355	12.09	297
Royalblue	9.09E−69	4.79E−70	138	4.70	73
Darkturquoise	3.82E−40	3.02E−41	134	4.56	52
Yellowgreen	4.24E−13	4.46E−14	52	1.77	18
Saddlebrown	6.48E−05	8.52E−06	88	3.00	13

**TABLE 2:** The miRNA under enriched modules.

**Module name**	**FDR**	***P*-value**	**Module size**	**Expected number of miRNAs**	**Number of miRNAs**
Gray	1.53E−23	4.04E−25	2933	99.91	20
Green	1.14E−15	5.98E−17	1397	47.59	4
Blue	3.76E−11	2.97E−12	1895	64.55	19
Black	5.19E−10	5.47E−11	1048	35.70	5
Yellow	7.32E−10	9.64E−11	1523	51.88	14
Red	1.44E−09	2.27E−10	1233	42.00	9
Brown	4.65E−09	8.57E−10	1862	63.43	23
Magenta	6.11E−07	1.29E−07	737	25.10	4
Greenyellow	9.62E−06	2.28E−06	583	19.86	3
Purple	0.000178	5.12E−05	685	23.33	7
Lightgreen	0.000178	5.14E−05	283	9.64	0
Lightcyan	0.000289	9.14E−05	340	11.58	1
Cyan	0.000618	0.000211	375	12.77	2
Salmon	0.00230	0.000846	432	14.72	4
Gray60	0.00261	0.00103	322	10.97	2
Darkgreen	0.0203	0.00854	137	4.667	0
Darkgray	0.0235	0.0105	131	4.462	0

### Analysis of miR-15a and miR-24-1 Targeting Genes in Co-expression Modules

**FIGURE 4:**
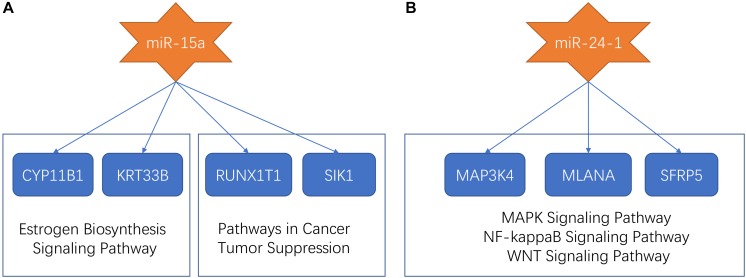
The dysfunctional pathways of miR-15a and miR-24-1. **(A)** The dysfunctional pathways of miR-15a. The miR-15a targeted CYP11B1, KRT33B, RUNX1T1, and SIK1. CYP11B1 and KRT33B were involved in the estrogen biosynthesis signaling pathway and RUNX1T1 and SIK1 were involved in pathways in cancer and tumor suppression process. **(B)** The dysfunctional pathways of miR-24-1. The miR-24-1 targeted MAP3K4, MLANA, and SFRP5 which participated in the MAPK signaling pathway, NF-kappaB Signaling pathway, and WNT signaling pathway, respectively.

Previous research proposed that miR-15a and miR-24-1 were oncogenic molecules which were associated with a poor prognostic of patients with ependymomas (Braoudaki et al., 2016). In our study, miR-15a and miR-24-1 were clustered into the midnight-blue module and saddle-brown module, respectively. Predicted target sites were collected from several databases to seek genes associated with miRNAs. We found four mRNAs (CYP11B1, KRT33B, RUNX1T1, and SIK1), which were targeted by miR-15a, and were co-expressed in the midnight-blue module. After applied annotation by the GeneCards database^9^ (Stelzer et al., 2016), we found that CYP11B1 and KRT33B were involved in the estrogen biosynthesis signaling pathway, while RUNX1T1 and SIK1 were involved in pathways in cancer and tumor suppression process. Three miR-24-1 targeting mRNAs (MAP3K4, MLANA, and SFRP5) were identified in the saddle-brown module. Functional annotation in the GeneCards database showed that such three mRNAs participated in MAPK signaling pathway, NF-kappaB Signaling pathway, and WNT signaling pathway, respectively, indicating that signaling pathways related to tumorigenesis and development were affected by miR-24-1. The dysfunctional pathways of miR-15a and miR-24-1 were summarized in Figure 4.

We checked the association of miR-15a and miR-24 with survival in 21 TCGA cancers (bladder carcinoma, breast cancer, cervical squamous cell carcinoma, esophageal adenocarcinoma, esophageal squamous cell carcinoma, head-neck squamous cell carcinoma, kidney renal clear cell carcinoma, kidney renal papillary cell carcinoma, liver hepatocellular carcinoma, lung adenocarcinoma, lung squamous cell carcinoma, ovarian cancer, pancreatic ductal adenocarcinoma, pheochromocytoma and paraganglioma, rectum adenocarcinoma, sarcoma, stomach adenocarcinoma, testicular germ cell tumor, thymoma, thyroid carcinoma, and uterine corpus endometrial carcinoma) using KM-plotter^10^ (Nagy et al., 2018). In 13 cancers (bladder carcinoma, cervical squamous cell carcinoma, esophageal adenocarcinoma, head-neck squamous cell carcinoma, liver hepatocellular carcinoma, lung squamous cell carcinoma, pancreatic ductal adenocarcinoma, pheochromocytoma and paraganglioma, rectum adenocarcinoma, sarcoma, stomach adenocarcinoma, thymoma, and uterine corpus endometrial carcinoma), the log rank *p*-values of miR-15a were smaller than 0.05. In eight cancers (bladder carcinoma, cervical squamous cell carcinoma, kidney renal clear cell carcinoma, liver hepatocellular carcinoma, rectum adenocarcinoma, sarcoma, stomach adenocarcinoma, and uterine corpus endometrial carcinoma), the log rank *p*-values of miR-24 were smaller than 0.05. Both miR-15a and miR-24 played important roles in various cancers. The findings we discovered may also be helpful in understanding the mechanisms of other cancers.

## Discussion

Genes (mRNAs) and microRNAs (miRNAs) were identified as molecular markers for clinical management based on the change of expression signatures in ependymoma (Costa et al., 2011; Wani et al., 2012). However, interaction mechanisms between miRNA and mRNA, as well as between co-expression networks, remain unclear. Apart from previous studies that focus on differentially expressed genes (Dai et al., 2017) or genes associated with disease, e.g., genes with driver mutations (Liang et al., 2017), whole gene sets containing totally 20,502 mRNAs and 723 miRNAs were analyzed without any filtration in the current study. In this manner, on the one hand, analysis can be performed independent of clinical grouping or unsupervised grouping. On the other hand, it is beneficial for detecting formerly neglected genes with low expression levels or little fluctuation between groups.

We used high-performance computers to construct a co-expression network of 64 EPN samples with both mRNA and miRNA profiles. The network model passed strict and reasonable soft-threshold criteria to meet approximate scale-free topology. In our results, a total of 18,292 genes were clustered into 37 modules with intrinsic association (excluding 2933 genes in gray modules without association). We found that more than 80% of modules (31 out of 37) included miRNA, indicating an extensive participation of miRNA in the regulation of gene co-expression networks. Considering the complexity of tumor cell biological process, it is insufficient to investigate the pathogenesis of tumors from a single type of RNA alone, while analyzing co-expression profiles of integrated miRNA and mRNA pairs could identify the regulatory network for a prognosis of brain tumors (Bing et al., 2016).

Subsequently, we performed functional enrichment analysis on clustered modules to determine gene groups that were intrinsically related to the progress of EPN. Interestingly, KEGG enrichment analysis showed that the most significant pathway was the cell cycle pathway in the red module, which was closely related to various intracellular molecular activities and cell proliferation capabilities. Development of the nervous system and brain evolution were affected by changes in the cell cycle of neural stem cells, which, with an out-of-control subpopulation growth, may transform into initial brain tumors in a cancer stem cell niche (Singh et al., 2004; Salomoni and Calegari, 2010). The second most significant enrichment in the red module was the DNA replication pathway. Change in expression levels of genes involved in the DNA replication pathway directly reflected increased cell proliferation and division activity, implying that cells have more vigorous growth and proliferation capabilities, which were hallmarks characteristic unique to tumor cells.

Consistent with previous reports that the Synaptic vesicle cycle pathway is a key pathway for up-regulation in EPN (Zhong et al., 2018), this study found that the Synaptic vesicle cycle pathway was the most significantly enriched pathway in the turquoise module, which contains the largest amount of genes. Key tumorigenic molecules contained in the Synaptic vesicle cycle pathway, such as RAB3a, was found to accelerate tumor formation and promote tumor cell proliferation by its increased expression level (Kim et al., 2014). In addition, we also found genes highly enriched in Glutamatergic synapse terms that reported to drive growth and invasion of brain tumors by communicating between neurons and tumor cells (Venkataramani et al., 2019). Furthermore, the GABAergic synapse pathway was proposed to confer cell proliferation advantages in the neural microenvironment (Neman et al., 2014). Three such significant enrichment pathways contained in turquoise in our results indicated that this is a functional gene group that can cooperate to promote conversion of brain nerves into brain tumor cells. Gene ontology analysis validated the above results that the turquoise module was an important component of neuron and synapse synthesis and communication processes, while the red module contained a set of genes with encoding proteins to bind with DNA to regulate mitotic cell cycle process.

As we all know, miRNA play a role in regulating mRNA expression through initiating transcriptional repression and introducing mRNA cleavage or degradation. We identified five miRNA-enriched modules (>10%), including oncogenic molecules miR-15a and miR-24-1, which were previously reported as poor prognosis biomarkers in child patients with ependymoma (Braoudaki et al., 2016). Four target genes of miR-15a predicted from the miRNA database were identified in the midnight-blue module (CYP11B1, KRT33B, RUNX1T1, and SIK1). We found CYP and KRT were involved in estrogen synthesis and signaling pathway which, with an abnormal status, was considered to be inseparable from the occurrence of multiple types of cancer (Chen et al., 2008; Gallo et al., 2010; Germain, 2011). Although this signal pathway has not yet been proposed to participate in brain tumors, functional disorders happening on cell-specific estrogen synthesis pathways in the brain can also cause brain diseases (Cui et al., 2013). Our results predict that miR-15a may delay the aging process of nerve cells by regulating a signal pathway which has never been discovered before, thereby promoting nerve cells development to malignant proliferating cells. RUNX1T1 has the ability to inhibit the growth of tumor cells (Alfayez et al., 2016) and can be used to predict cancer metastasis based on its reduced expression (Nasir et al., 2011). Researchers considered RUNX1T1 as a prognostic biomarker for CNS tumors (Liang et al., 2017). Based on our findings in co-expression networks, expression interaction of miR-15a and RUNX1T1 pair will lead to cancer transcriptional disorders, as well as encourage tumor cell growth and invasion. Inactivated SIK1 were reported to damage TP53-dependent anoikis which endow tumor cells with metastatic proficiency (Cheng et al., 2009); we infer that interaction between miR-15a and SIK1 may be a potential cause of uncontrolled growth of ependymal tumor cells.

Three target genes (MAP3K4, MLANA, and SFRP5) of miR-24-1 were detected in the co-expression network module (saddle-brown). After annotation, we found that they affected the development progress of tumors through three different signaling pathways. Firstly, MAP3K4 was involved in MAPK kinase signal transduction, which was a characteristic signaling pathway for discriminating group A subtype ependymoma (Archer and Pomeroy, 2011). Secondly, MLANA as a marker for the proliferation of melanocytes can be used to reflect brain-specific signatures of melanoma metastasis (Soikkeli et al., 2007; Nygaard et al., 2014). Involvement of MLANA in the regulation of NF-kappaB signaling pathway drives specific immuno-phenotype in group A ependymoma (Griesinger et al., 2017). Thirdly, SFRP5 participated in the WNT signaling pathway to regulate cell proliferation, migration, and cell fate decision. Dysregulation of WNT signaling was associated with various solid tumors, including glioblastoma (Lee et al., 2016). The expression interaction between miR-24-1 and these three genes detected in the current study reflected the potential expression regulation of MAPK, NK-kappaB, and WNT signaling pathways in ependymal tumor cells, suggesting that miR-24-1 promoted tumor progression by targeting genes on important signaling pathways that are closely related to cell proliferation and migration.

## Conclusion

To summarize, based on a weighted gene co-expression network approach, we identified enriched biological processes and pathways composed of associated genes that were related to ependymoma development. Our study reveals that, for the first time, the key regulatory mechanism of miRNAs is in promoting tumorigenesis and tumor development by analyzing co-expression network of miRNAs and mRNAs in ependymoma. Discoveries in the current study not only cover unexplored molecular mechanisms of miRNAs serving as a prognostic biomarker, but also propose novel genes that can be used for diagnosis signature and for potential antitumor treatment targets of ependymoma.

## Data Availability Statement

Publicly available datasets were analyzed in this study. This data can be found here: https://www.ncbi.nlm.nih.gov/geo/query/acc.cgi?acc=GSE21687.

## Author Contributions

TH designed the experiment. FL and HD performed the experiment and analyzed the data. HD and ZM wrote the manuscript.

## Conflict of Interest

The authors declare that the research was conducted in the absence of any commercial or financial relationships that could be construed as a potential conflict of interest.

## Footnotes

1 http://geneontology.org/

2 https://www.genome.jp/kegg/

3 http://www.microrna.org/microrna/

4 https://pictar.mdc-berlin.de/

5 http://www.microrna.gr/tarbase

6 http://mirdb.org/

7 http://www.mirbase.org/

8 http://www.targetscan.org

9 https://www.genecards.org/

10 http://kmplot.com/analysis/

## Supplementary Material

TABLE S1 The members of each module.

TABLE S2 The KEGG, GO BP, GO MF and GO CC enrichment FDR of each module.

TABLE S3 The overlap significance (hypergeometric test FDR) between our modules and the modules reported by Radulescu et al.

TABLE S4 The members of each module in mouse ependymoma model.

TABLE S5 The overlap significance (hypergeometric test FDR) between our modules and the modules in mouse ependymoma model.
